# Beyond a Single Continuum: Latent Profiles of Flourishing and Burnout Among Chinese Teachers

**DOI:** 10.3390/bs16101850

**Published:** 2026-10-09

**Authors:** Zhaoying He, Ziyao Liu, Disi Liang, Yujie Bai, Tongao Zeng, Jiaxiang Fan, Han Zhu

**Affiliations:** 1 Graduate School of Education, Peking University, Beijing 100871, China; zyhe2025@pku.edu.cn; 2 Faculty of Education, East China Normal University, Shanghai 200062, China; freizliu@stu.ecnu.edu.cn (Z.L.); 52274107002@stu.ecnu.edu.cn (T.Z.); 3 Faculty of Education, Beijing Normal University, Beijing 100875, China; liangds@mail.bnu.edu.cn (D.L.); byj@mail.bnu.edu.cn (Y.B.); 4 School of Marxism, Chongqing University, Chongqing 400044, China

**Keywords:** teacher flourishing, teacher burnout, latent profile analysis, occupational well-being, educational psychology

## Abstract

Teacher flourishing and burnout have often been viewed as opposing work experiences, implying that higher levels of one should be accompanied by lower levels of the other. However, recent research suggests that these experiences can coexist within the same individual. We extend this line of research by using latent profile analysis to examine how multiple dimensions of flourishing and burnout combine in different ways among 5694 primary and secondary school teachers in China. Five profiles were identified: Broadly Adaptive (61.19%), Struggling (19.41%), Engaged–Strained (8.96%), Unfulfilled (6.29%), and Depleted–Disconnected (4.16%). Broadly Adaptive and Struggling teachers followed the expected inverse pattern between flourishing and burnout. In contrast, Engaged–Strained teachers maintained moderate levels of flourishing and relatively high personal accomplishment despite substantial emotional exhaustion and depersonalization; Unfulfilled teachers showed favorable flourishing and low exhaustion and depersonalization but extremely low personal accomplishment; and Depleted–Disconnected teachers showed particularly poor emotional and social well-being alongside elevated exhaustion. These findings advance our understanding of teacher flourishing and burnout beyond a single-continuum model, although sensitivity analyses indicated that the retained profile solution was sensitive to the composition of the personal accomplishment measure, particularly for the Struggling and Unfulfilled profiles. Rather than asking simply whether teachers are flourishing or struggling with burnout, schools may benefit from asking in what areas teachers are feeling or functioning well and in what areas they are struggling.

## 1. Introduction

Teachers navigate increasing accountability pressures, changing professional demands, and complex interpersonal conditions. How they function under these conditions has consequences beyond their own occupational experience. Positive professional functioning has been associated with better teaching, stronger student relationships, higher student achievement, and a greater likelihood of remaining in the profession. Dysfunction, by contrast, has been associated with poorer classroom performance, absenteeism, and intentions to leave (Hascher & Waber, 2021; Madigan & Kim, 2021; Viac & Fraser, 2020; Zhou et al., 2024). Understanding teachers’ positive functioning and dysfunction in education settings is therefore important for sustaining a capable and healthy teaching workforce.

Positive professional functioning refers to a state of optimal well-being at work, encompassing not only favorable affective experiences, such as job satisfaction and positive affect, but also effective psychological functioning, including competence, meaning, and engagement, as well as positive social functioning characterized by connection, contribution, and integration within the workplace (Keyes, 2002; Seligman, 2018). Scholars have conceptualized such a state as flourishing at work (Rautenbach & Rothmann, 2017; Rothmann, 2013). By contrast, job burnout often captures the dysfunctional side of professional functioning, characterized by emotional exhaustion, depersonalization, and reduced personal accomplishment (Maslach et al., 2001; Maslach & Leiter, 2016).

Traditionally, flourishing and burnout have often been viewed as opposite ends of a single continuum, indicating that a higher level in one variable often means a lower level in the other. Consistent with this view, empirical evidence has documented a well-established negative association between these two variables (e.g., Carvalho et al., 2021; Freire et al., 2020; Vetter et al., 2018; for a review, see Zhou et al., 2024). However, an emerging body of research has suggested that the relationship between flourishing and burnout may be far more complex than a simplistic single-continuum model implies. For example, Moeller et al. (2018) found that some employees were highly engaged in their work while at the same time experiencing high levels of burnout. Rink et al. (2023) found that nurses who reported moderate-to-high levels of thriving could simultaneously experience high emotional exhaustion. R. S. Lee et al. (2021) found that medical faculty described deriving substantial meaning, growth, and satisfaction from their work while also experiencing considerable stress and emotional exhaustion. These findings challenge the single-continuum model of flourishing and burnout, suggesting that employees’ flourishing and burnout at work may be better understood through a more differentiated framework.

The inconsistent findings in prior research may arise from two possible reasons. First, both flourishing and burnout are multidimensional constructs comprising distinct but related components. These components need not occur at comparable levels within the same individual. A person may function well in some domains of flourishing but less well in others, just as different symptoms of burnout may vary within the same person (Bujacz et al., 2020; Leiter & Maslach, 2016). Treating flourishing and burnout as unitary constructs may therefore obscure meaningful heterogeneity in how their respective components are related. An overall negative association between the two constructs does not necessarily imply uniformly negative associations across all of their constituent dimensions.

Second, scholars have argued that well-being and ill-being may be better understood as two related but distinct continua rather than opposite ends of a single continuum (e.g., Keyes, 2002, 2005). From this perspective, an individual’s position on one continuum does not determine their position on the other. A person may simultaneously fall at the low or high ends of both continua, for example, experiencing both high well-being and high ill-being. Applied to the work context, this perspective helps explain why some employees can simultaneously experience high or low levels of both flourishing and burnout.

Accordingly, the present study aimed to advance our understanding of teacher flourishing and burnout by addressing these two issues. First, we explicitly conceptualized flourishing and burnout as multidimensional constructs composed of distinct but related components. Further, drawing on the two-continuum model of well-being (Keyes, 2002), we treated flourishing and burnout as separate continuums of professional functioning and examined how their components are configured within teachers. Our primary purpose was to identify subgroups of teachers characterized by distinct configurations of flourishing and burnout and to examine which demographic factors predict teachers’ membership in these subgroups.

### 1.1. Conceptual Models of Flourishing and Burnout

Building on Keyes’s model of mental health, Rothmann (2013) extended flourishing to work and defined it through emotional, psychological, and social well-being. Emotional well-being describes positive experiences and evaluations of work, including job satisfaction and positive affect. Psychological well-being encompasses autonomy, competence, relatedness, meaning, purpose, engagement, and learning at work. Social well-being concerns perceived functioning in the organizational community, including contribution, integration, acceptance, actualization, and coherence. Flourishing therefore includes both feeling well and functioning well psychologically and socially at work. Psychometric research has supported a multidimensional structure in which emotional, psychological, and social well-being are strongly related but empirically distinguishable dimensions (Joshanloo & Jovanović, 2017; Rautenbach & Rothmann, 2017). A recent systematic review likewise described flourishing as a holistic construct comprising multiple forms of positive functioning rather than a unitary psychological state (Peethambaran & Naim, 2025).

Burnout represents a distinct aspect of professional functioning. Maslach and colleagues conceptualized it as a work-related syndrome comprising emotional exhaustion, depersonalization or cynicism, and reduced personal accomplishment or professional efficacy (Maslach et al., 2001; Maslach & Leiter, 2016). Emotional exhaustion reflects depleted emotional and energetic resources. Depersonalization involves psychological distancing and detached or impersonal responses toward recipients of one’s work. Reduced personal accomplishment reflects diminished perceptions of professional competence and achievement. Although related, these dimensions capture distinguishable experiences rather than interchangeable manifestations of overall burnout. The Maslach Burnout Inventory (MBI) developers explicitly recommended that these three dimensions be interpreted separately (Maslach et al., 2018). Psychometric evidence showed that although the three dimensions are correlated, their items are not adequately explained by a single latent variable but instead form three distinguishable latent factors (Worley et al., 2008). Furthermore, the three dimensions have been associated with different antecedents and outcomes (R. T. Lee & Ashforth, 1996). Person-centered studies based on the Maslach framework have identified profiles dominated by exhaustion, cynicism, or inefficacy rather than uniform elevation across all dimensions (Leiter & Maslach, 2016). Comparable heterogeneity has been observed among teachers (Răducu & Stănculescu, 2022).

Together, flourishing and burnout may be better understood as constructs comprising related but distinct dimensions. Their multidimensional structures justify examining how these dimensions combine within teachers to form different subgroups.

### 1.2. Applying the Two-Continua Model to Flourishing and Burnout

Traditionally, mental well-being and mental illness were viewed as opposite ends of a single bipolar continuum (Keyes, 2005). The two-continua model of mental health challenged the assumption that positive mental health is defined solely by the absence of mental illness, arguing that positive mental health and mental illness instead constitute related but distinct constructs (Keyes, 2002, 2005). Empirical evidence supported this model, showing that people may experience high well-being along with high distress (Keyes, 2005). Applying the two continua model to the occupational domain, we expected that positive professional functioning (e.g., flourishing at work) does not preclude psychological difficulties (e.g., burnout).

Evidence from person-centered research provides empirical reasons for such expectation. Moeller et al. (2018) showed that high engagement could coexist with substantial burnout despite an overall negative association between the two constructs. Among teachers, Salmela-Aro et al. (2019) identified both a highly engaged profile and an engaged-burnout profile, with the latter combining considerable work engagement with emerging burnout symptoms. Subsequent studies have likewise identified heterogeneous combinations of engagement and burnout among teachers (Drouin-Rousseau et al., 2024; Holmström et al., 2023). Heterogeneity also occurs within burnout itself. Leiter and Maslach (2016) identified profiles in which exhaustion, cynicism, or inefficacy appeared without uniformly high levels of the other burnout dimensions. Latent profile analyses have identified groups with low exhaustion and depersonalization but low personal accomplishment, as well as groups dominated by exhaustion or cynicism (Răducu & Stănculescu, 2022).

Much of this literature, however, represents positive functioning through work engagement, which primarily captures motivational involvement and energetic investment. Engagement is an important indicator of flourishing but does not encompass its broader emotional, psychological, and social domains. Teachers may remain engaged while differing substantially in job satisfaction, meaning, or autonomy (Bujacz et al., 2020). In addition, profiles derived from burnout alone reveal heterogeneity within dysfunction but cannot show how these patterns coexist with flourishing. It therefore remains unclear whether multiple flourishing indicators and burnout symptoms form meaningful configurations when modeled simultaneously. Further person-centered analysis is thus needed to address this question.

Accordingly, drawing on Keyes’s (2002, 2005) two-continua model, the present study conceptualized flourishing and burnout as two related but distinct continua of work experience and examined how they are configured across different groups of teachers. This approach extends previous research by moving beyond a single-continuum framework. At the same time, we represented flourishing in terms of emotional, psychological, and social domains. This broader representation allows us to examine whether teachers may function well in some domains while experiencing difficulties in others, and whether these different aspects of flourishing can coexist with different symptoms of burnout.

### 1.3. The Present Study

The present study examined heterogeneity in teachers’ occupational well-being by considering flourishing and burnout simultaneously as multidimensional constructs. Specifically, we retained emotional, psychological, and social well-being as distinct dimensions of flourishing and emotional exhaustion, personal accomplishment, and depersonalization as distinct dimensions of burnout. Using latent profile analysis (LPA), a person-centered approach that identifies subgroups of individuals with similar patterns across multiple continuous measures, we examined how these six dimensions were configured within teachers. Building on the two-continua model, we expected that some teachers might display configurations that could not be readily ordered along a single continuum from positive (i.e., flourishing) to negative (i.e., burnout) occupational well-being. To this end, we addressed the following research questions (RQs):

RQ1: What distinct profiles of teachers can be identified based on the joint configuration of emotional, psychological, and social well-being and emotional exhaustion, personal accomplishment, and depersonalization?

RQ2: How are teachers’ demographic characteristics associated with the likelihood of membership in the identified flourishing and burnout profiles?

## 2. Materials and Methods

### 2.1. Participants and Procedure

This cross-sectional study collected online survey data from July to September 2025. Participants were recruited through online channels using convenience sampling and completed the survey through Wenjuanxing, a platform widely used for academic research in China. After providing electronic informed consent, participants reported demographic information and completed measures of flourishing and burnout. Mandatory response settings resulted in complete data for all submitted questionnaires. A total of 5849 questionnaires were submitted. Records were classified as exact duplicate submissions when responses were identical across all 32 scale items and six demographic variables. This criterion identified 278 records in 126 duplicate sets. Within each set, the first record in the original data file was retained and the remaining records were removed, resulting in the exclusion of 152 submissions. We additionally excluded three records for which age minus teaching experience was less than 13 years, implying that teaching would have begun before age 13. This was logically implausible. Completion-time screening was not performed because completion-time information was not available in the dataset provided for the present analysis. Straight-line responding, defined as selecting the same option across all 32 scale items, was observed in 75 records (1.32%). Because uniform responding alone cannot determine that responses are invalid, these records were retained in the primary analysis. The final analytic sample comprised 5694 teachers, including 2053 men (36.1%) and 3641 women (63.9%). Mean age was 37.01 years (*SD* = 9.55), and mean teaching experience was 14.07 years (*SD* = 10.08). Average class size was 46.52 students (*SD* = 9.22). Participants taught in primary (38.7%), junior high (33.8%), and senior high (27.4%) schools located in urban (30.2%), suburban (57.0%), and rural (12.8%) areas. Sample selection and full demographic characteristics are provided in Supplementary Materials, Table S1.

### 2.2. Measures

#### 2.2.1. Flourishing

Flourishing was measured with the Flourishing-at-Work Scale-Short Form (FAWS-SF; Rautenbach, 2015; Rautenbach & Rothmann, 2017). The Chinese version used in the present study was developed by the research team based on the original English instrument. Two bilingual members of the research team with experience in educational and psychological research independently translated the original English items into Chinese. The translated items were then compared carefully with the original wording to ensure consistency in meaning and conceptual content. An expert familiar with educational and psychological research subsequently reviewed the Chinese wording for semantic clarity and contextual appropriateness for Chinese teachers, and minor revisions were made where necessary.

The FAWS-SF contains 17 items assessing emotional well-being, psychological well-being, and social well-being. Emotional well-being (EW) includes three items: job satisfaction, positive affect, and gratitude. Psychological well-being (PW) includes nine items: autonomy, competence, relatedness, meaning, purpose, cognitive engagement, emotional engagement, physical engagement, and learning. Social well-being (SW) includes five items: social contribution, social acceptance, social actualization, social integration, and social coherence. Respondents rated how often they had experienced each item during the previous month on a five-point scale from 1 (never) to 5 (every day). Internal consistency was good for each subscale (Cronbach’s α: EW = 0.83; PW = 0.90; SW = 0.86). Confirmatory factor analysis using WLSMV estimation supported a three-factor model, χ^2^(116) = 3960.48, CFI = 0.970, TLI = 0.965, RMSEA = 0.076, 90% CI [0.074, 0.078], and SRMR = 0.031. Item allocation, reliability estimates, standardized factor loadings, and the complete CFA fit indices are provided in Supplementary Materials, Table S2.

#### 2.2.2. Burnout

Burnout was measured with the Maslach Burnout Inventory-Educator Survey (MBI-ES; Maslach et al., 1996). We used a cross-cultural version validated among Chinese teachers by Aboagye et al. (2018). The intended questionnaire contained five emotional exhaustion (EE) items (e.g., “I feel emotionally drained from my work”), seven personal accomplishment (PA) items (e.g., “I have accomplished many worthwhile things in this job”), and four depersonalization (DP) items (e.g., “I don’t really care what happens to some students”). During questionnaire preparation, one PA item of the original scale, “I feel very energetic,” was inadvertently omitted. The administered measure therefore contained 15 items including five EE items, six PA items, and four DP items, all scored from 1 (never) to 5 (always). Internal consistency for the current measurement was good (Cronbach’s α: EE = 0.89; PA = 0.86; DP = 0.87). Confirmatory factor analysis using WLSMV estimation provided mixed evidence regarding the fit of the three-factor model, χ^2^(87) = 6849.12, CFI = 0.955, TLI = 0.946, RMSEA = 0.117, 90% CI [0.115, 0.119], and SRMR = 0.073. Although CFI, TLI, and SRMR were relatively favorable, the elevated RMSEA indicated limitations in the fit of the specified three-factor model. Owing to the omission of one PA item, scores on the resulting six-item subscale are not directly comparable to scores or published norms based on the complete seven-item subscale. Scoring details and the complete measurement results for the administered burnout measure are provided in Supplementary Materials, Table S2.

#### 2.2.3. Demographic Characteristics

Participants reported gender, age, average class size, and years of teaching service. They also reported teaching level (primary, junior high, or senior high school) and school location (urban, suburban, or rural).

### 2.3. Statistical Analysis

#### 2.3.1. Latent Profile Analysis

LPA was conducted in Mplus version 8.3 using six continuous composite indicators: EW, PW, SW, EE, PA, and DP. All indicators were standardized before analysis, and models with one to seven classes were estimated using robust maximum likelihood. Because assumptions about within-class variances and covariances in LPA can affect class enumeration and the resulting profile structure (Masyn, 2013), we compared six alternative specifications that crossed equal versus class-specific variances with zero, targeted, and fully estimated within-class covariance structures (Table 1). Convergence and admissibility, statistical fit, and substantive interpretability were used to determine the most appropriate specification.

The broad enumeration runs used 2000 random sets of starting values, with 500 retained for final optimization and 100 initial-stage iterations. The five-, six-, and seven-class solutions were subsequently re-estimated with 5000 initial and 1000 final-stage starts to assess numerical reproducibility of the optimization and reduce the risk of retaining a local maximum. The selected solution for each class count was required to terminate normally and reproduce the best loglikelihood.

Model selection considered the Akaike information criterion (AIC), Bayesian information criterion (BIC), sample-size-adjusted BIC (aBIC), entropy, the Lo–Mendell–Rubin adjusted likelihood ratio test (LMR-LRT), bootstrap likelihood ratio test (BLRT), and substantive interpretability. Lower information criteria indicated better fit, and significant likelihood ratio tests indicated improvement over a model with one fewer class. Entropy values closer to 1 indicated clearer classification, with 0.80 used as a descriptive benchmark. A significant LMR-LRT and BLRT indicated that a model with K classes improved on the model with K − 1 classes.

Demographic predictors of profile membership were examined in Mplus 8.3 using the automatic R3STEP procedure. This approach estimated a multinomial logistic regression while correcting for uncertainty in assigning teachers to latent profiles. Categorical variables including gender, teaching level, and school location were dummy-coded. Continuous variables including age, class size, and teaching experience were standardized. Because age and teaching experience were highly correlated (*r* = 0.959), they were entered in separate models. We report the teaching experience model in the main text. The age model had similar results of the teaching experience model and can be found in Supplementary Materials, Table S7.

#### 2.3.2. Leave-One-Item-Out Sensitivity Analyses

Because one item from the PA subscale was inadvertently omitted during questionnaire preparation, we conducted a series of leave-one-item-out sensitivity analyses to assess the robustness of the identified profile solution to the composition of the administered PA subscale. In each of six analyses, one of the six administered PA items was omitted, PA scores were recalculated as the unweighted sum of the remaining five items, and all six profile indicators were restandardized within the same analytic sample. The LPA was then re-estimated using the variance–covariance specification and number of profiles retained in the primary analysis. After matching the resulting profiles to the primary solution, robustness was evaluated based on agreement in modal classifications, the adjusted Rand index (ARI), changes in model-estimated indicator means and profile proportions, and retention of the substantive profile configurations.

## 3. Results

### 3.1. Preliminary Analysis

Table 2 presents descriptive statistics and Pearson correlations for the six composite indicators. The flourishing dimensions were positively correlated (*r* = 0.69 to 0.76, all *p* < 0.001). Their correlations with EE and DP were negative (*r* = −0.18 to −0.29, all *p* < 0.001). PA correlated positively with flourishing (*r* = 0.26 to 0.36) and EE (*r* = 0.09), and negatively with DP (*r* = −0.06). EE and DP were positively correlated (*r* = 0.64).

### 3.2. Latent Profiles

#### 3.2.1. Selection of the Variance–Covariance Specification

The equal-variance specifications (S1, S3, and S5) resulted in admissible solutions across the class counts examined. Among the admissible specifications, the full-covariance specification (S3) substantially improved model fit compared to zero-covariance specification (S1) and partial-covariance specification (S5). We therefore selected S3 as the primary specification for subsequent class enumeration. Complete fit indices and admissibility results for all six specifications are provided in Supplementary Materials, Table S3.

#### 3.2.2. Model Selection

Table 3 presents the fit statistics for the one- through seven-class models. AIC, BIC, and aBIC decreased at every step. The elbow plot (see Supplementary Materials, Figure S1) showed that decreases in all information criteria tended to flatten after the four-class model. The entropy of the four-class model was higher than that of the five- and six-class models. The BLRT remained significant from the two- through seven-class models, whereas the LMR-LRT supported successive increases through six classes (*p* < 0.001) but did not support seven classes (*p* = 0.2257). Thus far, the fit indices seemed to support a four-, five-, or six-class solution. We further carefully examined what the higher-class models added from the four-class model to the six-class model.

**Figure 1:**
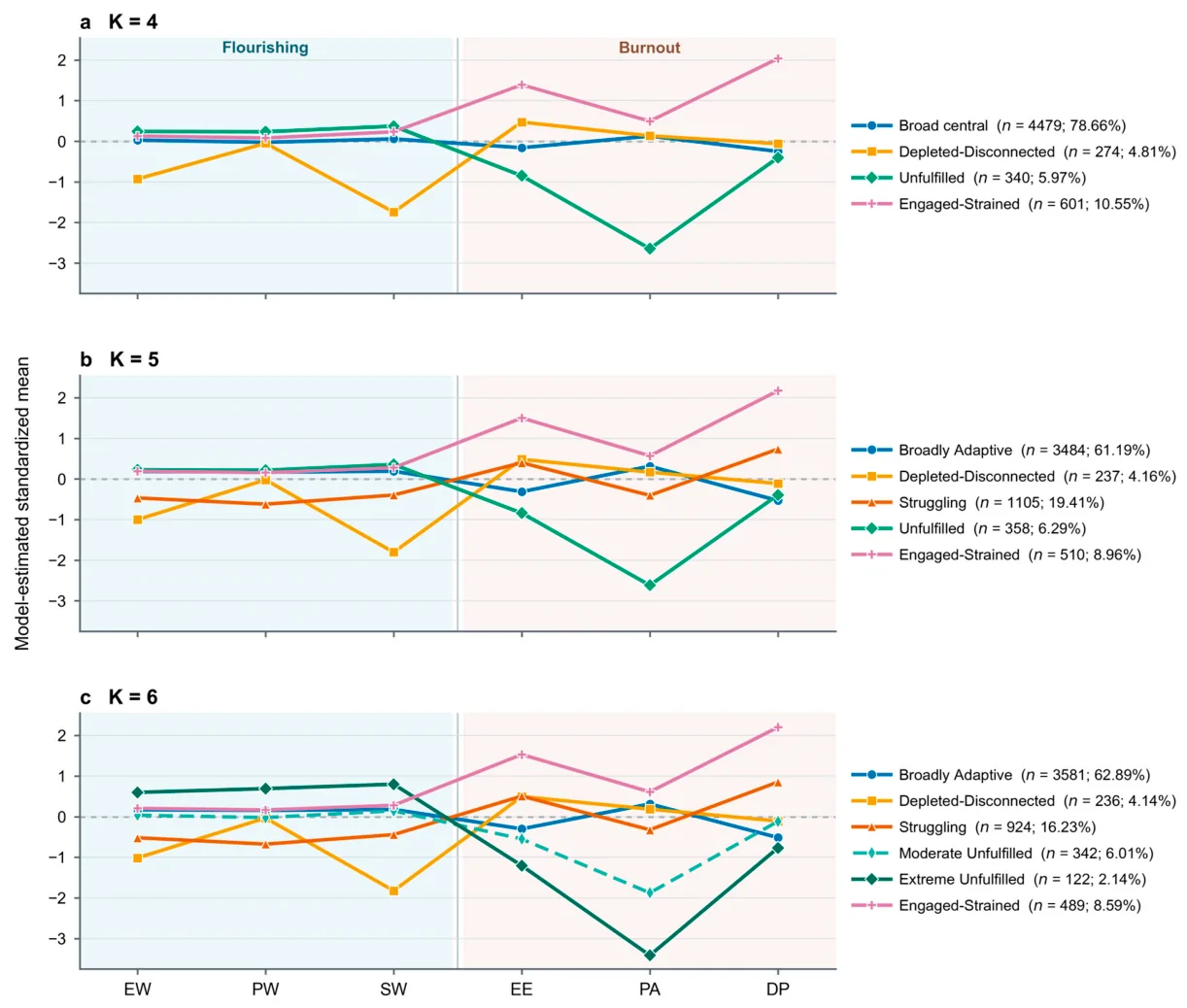
Profile structure across the four-, five-, and six-class models. Panels (**a**–**c**) show the model-estimated standardized indicator means for the four-, five-, and six-class solutions, respectively. The gray horizontal dashed line at zero represents the overall sample mean for each standardized indicator.

As depicted in Figure 1, moving from the four-class to the five-class model produced a substantively meaningful reorganization of the profile structure. The K4 Broad central class was divided primarily into the K5 Broadly Adaptive and Struggling profiles. Specifically, 77.32% of its members entered Broadly Adaptive, whereas 22.24% entered Struggling, and 90.14% of the K5 Struggling profile originated from this K4 class. The Unfulfilled, Depleted–Disconnected, and Engaged–Strained profiles were otherwise largely preserved. The six class model mainly subdivided the K5 Unfulfilled profile. Of the K5 Unfulfilled members, 65.92% entered a Moderate Unfulfilled class and 34.08% entered an Extreme Unfulfilled class at K6. Both descendants closely retained the shape of the K5 profile, with correlations of 0.988 and 0.990, respectively. The smaller descendant contained only 122 teachers, or 2.14% of the sample. Thus, K5 introduced a distinct and interpretable configuration that was not represented at K4, whereas K6 provided a finer quantitative distinction within an existing configuration rather than a qualitatively new pattern. The five class model was therefore retained as the final solution. Class correspondence and the similarity of the two K6 Unfulfilled descendants to their K5 source profile are detailed in Supplementary Materials, Table S5.

#### 3.2.3. Profile Descriptions

Figure 1b displays the model-estimated standardized indicator means for the retained five-class solution, and Table 4 provides the corresponding values. The model estimates a posterior probability for each teacher’s membership in each profile, representing the probability of that membership given the teacher’s observed indicator scores and the fitted model. For reporting sample sizes and percentages, each teacher was assigned to the profile with the highest posterior probability. The profile means were taken directly from the fitted latent class model.

**The Broadly Adaptive profile.** This profile was the largest group and included 61.19% of the sample (*n* = 3484). EW, PW, and SW were slightly above the sample mean, with standardized model-estimated means of 0.182, 0.168, and 0.191, respectively. EE and DP were below the mean (−0.313 and −0.533), while PA was modestly above the mean (0.313). This profile represented a broadly adaptive pattern characterized by moderately positive flourishing together with comparatively low burnout.

**The Depleted–Disconnected profile.** This profile included 4.16% of the sample (*n* = 237). EW was low (−1.001) and SW was especially low (−1.804), whereas PW was close to the sample mean (−0.022). EE was moderately elevated (0.484), while PA (0.167) and DP (−0.114) remained near the mean. The core feature of this group was the pronounced deficits in emotional and especially social well-being, accompanied by some emotional depletion.

**The Struggling profile.** This profile included 19.41% of the sample (*n* = 1105). All three flourishing indicators were below the mean (EW = −0.467, PW = −0.621, and SW = −0.396). EE and DP were elevated (0.399 and 0.737), and PA was low (−0.405). This profile combined broadly reduced flourishing with an unfavorable burnout configuration.

**The Unfulfilled profile.** This profile included 6.29% of the sample (*n* = 358). EW, PW, and SW were slightly above the mean (0.225, 0.214, and 0.356), and EE and DP were comparatively low (−0.841 and −0.395). PA, however, was markedly low (−2.618). Teachers in this profile appeared to retain generally positive emotional, psychological, and social functioning and showed little exhaustion or depersonalization, yet experienced a lack of personal accomplishment.

**The Engaged–Strained profile.** This profile included 8.96% of the sample (*n* = 510). Flourishing was near to slightly above the mean (EW = 0.193, PW = 0.157, and SW = 0.275), and PA was also above the mean (0.570). At the same time, EE was high (1.501) and DP was especially high (2.172). This configuration showed that moderate-to-high flourishing and perceived accomplishment can coexist with substantial exhaustion and interpersonal disengagement.

#### 3.2.4. Demographic Characteristics and Profile Membership

Table 5 presents the multinomial logistic regression results for the demographic predictors of profile membership. The results suggested that a one standard deviation increase in teaching years was associated with higher odds of Broadly Adaptive membership than Struggling membership, OR = 1.301, 95% CI [1.175, 1.440], *p* < 0.001, *q* < 0.001. Rural residence was associated with higher odds of Engaged–Strained membership than Depleted–Disconnected membership, OR = 3.955, 95% CI [1.778, 8.799], *p* < 0.001, *q* = 0.026.

#### 3.2.5. Robustness of the Five-Profile Solution

Five of the six PA item deletion analyses closely reproduced the primary solution. Omitting B4, B7, B10, B12, or B14 individually resulted in modal classification agreement of 97.44% to 98.35% and ARI values of 0.933 to 0.964, with all five substantive profile configurations retained. Omitting B13, however, substantially changed profile configurations and proportions. Modal classification agreement decreased to 76.24%, and the ARI decreased to 0.546. Following profile matching, the proportion assigned to Broadly Adaptive increased from 61.19% to 75.41%, whereas Struggling decreased from 19.41% to 7.11% and Unfulfilled decreased from 6.29% to 2.32%. The matched Broadly Adaptive and Struggling profiles did not retain their primary qualitative configurations. These results indicate that the retained solution was sensitive to the composition of the administered PA subscale. The identified profiles should therefore be regarded as provisional configurations, with particular caution warranted when interpreting Struggling and Unfulfilled. Detailed results are provided in Supplementary Materials, Tables S8–S11 and Figure S2.

## 4. Discussion

Prior research on flourishing and burnout has shown seemingly inconsistent findings. A substantial body of research has suggested that these two variables are generally negatively associated, such that elevated levels of one are typically accompanied by reduced levels of the other (Carvalho et al., 2021; Freire et al., 2020). But research has also shown that individuals who function positively at work can simultaneously experience substantial burnout (R. S. Lee et al., 2021; Moeller et al., 2018; Rink et al., 2023). The present study revisits this issue by employing LPA to identify distinct configurations of flourishing and burnout in a large sample of teachers. By doing so, we examine how different dimensions of flourishing and burnout combine in distinct ways within subgroups of teachers and explore whether demographic factors are associated with subgroup membership.

Our results suggested five provisional configurations of flourishing and burnout dimensions. Most teachers fell into the Broadly Adaptive or Struggling profiles, which followed the expected inverse pattern between flourishing and burnout. The former was characterized by relatively favorable flourishing and burnout scores, whereas the latter showed the opposite pattern. However, this inverse pattern did not characterize all teachers. The Engaged–Strained, Unfulfilled, and Depleted–Disconnected profiles showed discrepancies across specific dimensions of flourishing and burnout that could not be readily ordered along a single continuum from favorable to unfavorable functioning. These findings suggest that the well-established negative association between flourishing and burnout captures an important general tendency, but does not fully describe the diversity of teachers’ occupational experiences.

### 4.1. Patterns of Flourishing and Burnout Across Teacher Profiles

The Broadly Adaptive and Struggling profiles suggest that the conventional inverse pattern remains highly relevant for understanding flourishing and burnout. Together, these two profiles accounted for 80.6% of the sample, although sensitivity analyses indicated that both their prevalence and configuration changed when B13 was omitted from the PA measure, particularly for the Struggling profile. Teachers in the Broadly Adaptive profile showed somewhat more favorable flourishing, lower emotional exhaustion and depersonalization, and higher personal accomplishment, whereas the Struggling profile displayed the opposite configuration. This pattern is consistent with prior variable-centered evidence showing that flourishing is generally negatively associated with burnout (Vetter et al., 2018; Zhou et al., 2024). For a substantial proportion of teachers, positive and negative aspects of professional functioning did vary in the expected opposite directions. They either flourish at work and experience minimal burnout, or struggle with burnout alongside lower well-being across emotional, psychological, and social domains.

The results of regression analysis suggest that teachers with longer teaching experience were more likely to belong to the Broadly Adaptive profile than to the Struggling profile. One possible explanation is that accumulated professional experience may be accompanied by greater instructional competence, more established coping routines, and a clearer sense of how to manage recurring occupational demands. However, teaching experience may not always be a protective factor against burnout. Past profile research has found that teachers with more teaching experience are more likely to belong to a high burnout profile (e.g., Alghamdi & Sideridis, 2025). This suggests that the implications of career stage may depend on the specific configuration of demands, resources, and burnout dimensions.

Perhaps more importantly, we detected three profiles that fall outside the inverse pattern of flourishing and burnout. The Engaged–Strained profile represented 8.96% of the sample. Teachers in this profile reported relatively favorable emotional, psychological, and social well-being and relatively high personal accomplishment, while simultaneously showing the highest levels of emotional exhaustion and depersonalization. This pattern is consistent with previous person-centered research showing that positive involvement in work can coexist with substantial strain. Moreover, the particularly high levels of emotional exhaustion and depersonalization in this profile may help explain how substantial burnout can coexist with favorable professional functioning. Emotional exhaustion captures the energetic component of burnout and is thought to arise most directly from sustained workload and other demands that consume emotional resources (Maslach & Leiter, 2016). Teachers may therefore continue to experience meaning, competence, positive affect, and organizational belonging while nevertheless becoming emotionally depleted by the effort required to meet persistent professional demands. Depersonalization, on the other hand, represents a subsequent and more interpersonal response to such depletion. Classical models of burnout describe psychological distancing from recipients of one’s work as a defensive response to exhaustion, through which employees reduce further emotional involvement when their resources are strained (Maslach & Leiter, 2016). Consistent with this argument, longitudinal research among teachers has found that emotional exhaustion predicts subsequent increases in depersonalization (Park & Lee, 2013). Overall, our study identifies a subgroup of teachers who function positively (e.g., satisfied with job, engaged in task, and feel connected with the school) and perceive themselves as effective, but who may do so at the cost of emotional resource depletion and increasing psychological distance from students, potentially as a way of managing that depletion.

The Unfulfilled profile provides an informative contrast with the Engaged–Strained profile. These two groups had similar model-estimated means across the three flourishing dimensions but showed contrasting configurations of emotional exhaustion, personal accomplishment, and depersonalization. Engaged–Strained teachers reported high emotional exhaustion and depersonalization while maintaining above-average personal accomplishment. By contrast, Unfulfilled teachers exhibited below-average emotional exhaustion and depersonalization with extremely low personal accomplishment. This profile is consistent with prior person-centered research showing that professional inefficacy occurs without corresponding elevations in exhaustion or cynicism (Leiter & Maslach, 2016). Nevertheless, the Unfulfilled profile should be regarded as provisional because its identification was sensitive to the composition of the PA measure, which is particularly relevant given that one intended PA item was inadvertently omitted in the present study. If this configuration is replicated using the complete PA measure, the contrast between the Engaged–Strained and the Unfulfilled profiles may contribute to a more nuanced understanding of teacher flourishing and burnout. Teachers may not only simultaneously experience flourishing and substantial burnout, but that those displaying this broader pattern may in fact experience distinct forms of burnout (e.g., emotional exhaustion and interpersonal detachment versus low accomplishment). One possible explanation is that different burnout symptoms arise from different psychological and occupational mechanisms. Further research is needed to test this possibility.

This finding also suggests that the three symptoms of burnout should be examined in a more fine-grained way. Emotional exhaustion, depersonalization, and reduced personal accomplishment capture distinguishable aspects of employees’ relationship with their work and need not develop in parallel (Maslach & Leiter, 2016). The MBI developers explicitly recommend that the three dimensions be calculated and interpreted separately rather than combined into a single burnout score. But, a large body of prior research has represented burnout by aggregating the three symptoms into a composite score. Our findings prompt future research to carefully reconsider what such a composite score actually represents (see also Bianchi et al., 2024).

Finally, we identified a small group of teachers (4.16% of the sample) mainly characterized by emotional depletion and social dysfunction, namely, the Depleted–Disconnected profile. Teachers in this subgroup reported average levels of psychological well-being, near-average levels of personal accomplishment and depersonalization, alongside elevated emotional exhaustion, low emotional well-being, and extremely low social well-being. The particularly low social well-being in this profile may indicate a weakened sense of functioning within the school. In the flourishing-at-work framework, social well-being concerns experiences such as belonging, integration, contribution, acceptance, and coherence within the organization (Rothmann, 2013; Rautenbach & Rothmann, 2017). Teachers in this profile may therefore continue to feel competent, purposeful, and engaged in their work while feeling less connected to, integrated within, or supported by the broader school environment. This aligns with prior research showing that well-being can be uneven across domains, with social well-being often being lower than emotional and psychological well-being (Ford et al., 2025; Moller & Rothmann, 2019). At the same time, their low emotional well-being and moderately elevated exhaustion suggest that such social disconnection may coexist with poorer affective experiences and emotional depletion. Future research is needed to explore whether weak social functioning contributes to emotional depletion, whether exhaustion erodes teachers’ sense of connection to the school, or whether both arise from broader organizational conditions.

The results suggested that rural teachers (compared to urban teachers) were more likely to belong to the Engaged–Strained profile than to the Depleted–Disconnected profile. Although both profiles involved emotional exhaustion, Engaged–Strained teachers retained relatively high personal accomplishment and more favorable emotional and social well-being, whereas Depleted–Disconnected teachers showed poorer emotional and especially social well-being. This suggests that rural teachers are more likely to remain professionally effective and perceive themselves as contributing members of their schools. One possible explanation may be the particular combination of constraints and opportunities associated with rural teaching. Rural schools in China often operate with fewer educational and human resources, requiring teachers to undertake heavier teaching loads and a broader range of responsibilities. At the same time, rural teachers have been found to assume more important school duties, and research suggests that many derive a strong sense of professional value from their contribution to students and local education (Qin & Zeng, 2018). In such contexts, teachers may have greater responsibility and a more visible role in the functioning of the school. This may allow some teachers to experience competence, accomplishment, and contribution despite the substantial emotional costs of carrying these responsibilities. In other words, resource scarcity may simultaneously increase the demands placed on rural teachers and increase the perceived significance of what they contribute.

### 4.2. Implications for Practice

The current results have some implications for practice. First, our findings suggest that positive professional functioning should not be taken as evidence that negative functioning is absent. For example, teachers may report favorable emotional, psychological, and social well-being while simultaneously experiencing substantial burnout, as illustrated by the Engaged–Strained profile. Schools should therefore assess both the positive and negative aspects of teachers’ professional functioning instead of relying on either domain alone. Such assessment should also consider the multidimensional nature of both flourishing and burnout. Rather than asking simply whether teachers are doing well or poorly, schools may benefit from asking in what ways teachers are functioning well and in what ways they are struggling.

Second, the differentiated profiles also illustrate why attention to specific dimensions may matter in practice. Teachers resembling the Struggling profile showed difficulties across several aspects of both flourishing and burnout, whereas those resembling the Engaged–Strained profile maintained relatively favorable flourishing and personal accomplishment despite substantial exhaustion and depersonalization. The latter pattern suggests that visible indicators of positive functioning, such as engagement, satisfaction, or perceived accomplishment, should not be assumed to indicate an absence of occupational strain. Similarly, the Unfulfilled profile indicates that low exhaustion alone may provide an incomplete picture when teachers experience very little sense of professional accomplishment. The Depleted–Disconnected profile further suggests that difficulties in emotional and social well-being may be obscured when attention is concentrated primarily on general psychological functioning or conventional burnout measures. Taken together, these patterns suggest that schools may gain useful information by assessing teachers across multiple domains before determining what forms of support, if any, may be appropriate.

### 4.3. Limitations and Future Research

Several limitations of this study should be acknowledged. First, the cross-sectional design limits conclusions about the temporal development and stability of the identified profiles. The present study shows that teachers can display distinct configurations of flourishing and burnout at a given point in time, but it cannot determine whether these profiles represent relatively stable patterns or transient states. Future research should therefore use longitudinal designs, particularly latent transition analysis, to examine the stability of profile membership, transitions between profiles, and the occupational conditions that precede such transitions. Second, the use of online convenience sampling limits the representativeness and generalizability of the findings. Although the final sample was large, participation was voluntary and restricted to teachers who accessed the online survey, potentially underrepresenting teachers with limited digital access or skills and those whose workload or strain restricted participation. Future studies should replicate the profile structure using probability-based or nationally representative samples. Third, one intended PA item from the MBI-ES was inadvertently omitted during questionnaire preparation. Although the administered six-item PA subscale showed good internal consistency, the CFA fit indices provided mixed evidence for the specified three-factor measurement model, with an elevated RMSEA despite more favorable CFI and SRMR values. Because the profile indicators were unit-weighted subscale scores, this misfit implies that the EE, PA, and DP scores may contain variance not fully captured by the intended three-factor structure. Differences in these indicators across profiles are therefore better interpreted as differences in observed subscale scores than as precise contrasts between latent burnout dimensions. Sensitivity analyses indicated that the five-profile solution was largely preserved when each of five administered PA items was omitted individually, but changed substantially when B13 was omitted. This sensitivity to a single item warrants caution when interpreting profiles distinguished by PA, particularly Struggling and Unfulfilled. The concern is especially relevant to Unfulfilled, which was defined primarily by exceptionally low PA. Replication with the complete MBI-ES is needed to assess whether these profiles remain distinguishable when personal accomplishment is measured with the full item set. Fourth, the five-profile solution was obtained under the S3 specification, which constrained indicator variances and within-profile covariances to equality across profiles. Alternative specifications allowing class-specific variances did not converge adequately at the relevant class counts, limiting comparisons across variance–covariance assumptions. Although increasing the number of random starts reproduced the same maximum loglikelihood, this reflects numerical reproducibility of model optimization rather than validation of the profile structure. Thus, the current profile structure was obtained under the selected variance–covariance specification and has not been independently or split-sample validated. Future research should examine whether these configurations can be reproduced across alternative model specifications and independent samples. Finally, although the present study identified distinct configurations of flourishing and burnout among teachers, the quantitative design does not allow us to examine in depth how teachers themselves experience and interpret these configurations or the contextual processes through which they develop. Future research could adopt qualitative or mixed-methods designs to investigate the lived experiences, occupational conditions, and perceived processes underlying different profiles. Interviews with teachers representing different profiles may be particularly useful for clarifying how these configurations emerge and are sustained in everyday school contexts, thereby providing a more nuanced understanding of teacher professional functioning.

## 5. Conclusions

Using LPA in a large sample of teachers, the present study advances our understanding of teacher flourishing and burnout beyond the conventional single-continuum model. In our results, although most teachers belonged to the Broadly Adaptive or Struggling profiles, which followed the expected inverse pattern, three additional profiles revealed more differentiated configurations. Engaged–Strained teachers maintained moderate levels of flourishing and relatively high personal accomplishment while experiencing substantial emotional exhaustion and depersonalization. Unfulfilled teachers showed relatively favorable flourishing and low exhaustion and depersonalization but extremely low personal accomplishment. Depleted–Disconnected teachers were distinguished by particularly poor emotional and social well-being alongside elevated exhaustion. Because the retained solution was sensitive to the composition of the PA measure, these five profiles should be regarded as provisional configurations, particularly the Struggling and Unfulfilled profiles. Nevertheless, these patterns suggest that positive and negative aspects of teachers’ professional functioning can coexist, while different dimensions within flourishing and burnout may also diverge within the same teacher. Such heterogeneity may be obscured when flourishing and burnout are reduced to single overall scores. This matters in practice because teachers who appear to be functioning well in one domain may still experience substantial difficulty in another, and these difficulties may remain unnoticed when assessment relies primarily on broad indicators of well-being or burnout. Rather than relying on overall well-being or burnout scores, schools may gain a more informative picture of teachers’ professional functioning by identifying the specific subdomains in which teachers are functioning well and those in which they are struggling. Such assessment may help schools identify different forms of difficulty and better match support to teachers’ specific needs.

## Figures and Tables

**Table 1:** Variance and covariance specifications considered in the latent profile analysis.

Specification	Indicator Variances	Within-Profile Covariances	Convergence/Admissibility
S1	Equal across profiles	All fixed to zero	K1 through K7 admissible
S2	Freely estimated by profile	All fixed to zero	K1 and K2 admissible; K3 through K7 did not terminate normally and included negative variance
S3	Equal across profiles	All 15 freely estimated and constrained to equality across profiles	K1 through K7 admissible
S4	Freely estimated by profile	All 15 freely estimated by profile	K1 and K2 admissible; K3 through K7 did not terminate normally under the specified search settings
S5	Equal across profiles	Four targeted covariances freely estimated and constrained to equality across profiles; remaining 11 fixed to zero	K1 through K7 admissible
S6	Freely estimated by profile	Four targeted covariances freely estimated by profile; remaining 11 fixed to zero	K1 admissible; K2 through K7 did not terminate normally under the specified search settings

Note. The four targeted covariances in S5 and S6 are EW with PW, EW with SW, PW with SW, and EE with DP, because they are theoretically related. K means the number of classes.

**Table 2:** Descriptive statistics and Pearson’s correlations among flourishing and burnout indicators (*N* = 5694).

Variable	*M*	*SD*	1	2	3	4	5
1. EW	12.53	1.78					
2. PW	38.24	4.58	0.75				
3. SW	20.56	3.02	0.69	0.76			
4. EE	13.45	4.72	−0.29	−0.25	−0.25		
5. PA	23.26	4.31	0.29	0.36	0.26	0.09	
6. DP	8.40	3.86	−0.23	−0.28	−0.18	0.64	−0.06

Note. *M* = mean; *SD* = standard deviation. All correlation coefficients were significant at *p* < 0.001.

**Table 3:** Model-fit indices for one- through seven-class latent profile solutions under the selected S3 specification.

Model	Loglikelihood	AIC	BIC	aBIC	Entropy	LMR-LRT (*p*)	BLRT (*p*)
1-Class	−40,997.389	82,048.779	82,228.252	82,142.454			
2-Class	−39,837.015	79,742.031	79,968.035	79,859.993	0.972	<0.001	<0.001
3-Class	−38,888.741	77,859.481	78,132.015	78,001.729	0.944	<0.001	<0.001
4-Class	−38,346.389	76,788.778	77,107.842	76,955.312	0.928	<0.001	<0.001
**5-Class**	**−38** **,** **105.643**	**76** **,** **321.286**	**76** **,** **686.881**	**76** **,** **512.107**	**0.838**	**<0.001**	**<0.001**
6-Class	−37,855.704	75,835.407	76,247.532	76,050.514	0.859	<0.001	<0.001
7-Class	−37,641.693	75,421.387	75,880.041	75,660.780	0.871	0.2257	<0.001

Note. *N* = 5694. BLRT with 500 bootstrap samples. High-STARTS runs for the five-, six-, and seven-class models reproduced the same maximum loglikelihoods as the corresponding broad-search runs, indicating numerical reproducibility of the optimization under the selected S3 specification. Bold indicates the retained five-class solution.

**Table 4:** Model-estimated standardized means and modal class sizes for the five-class solution.

Profile	*n* (%)	EW	PW	SW	EE	PA	DP
Broadly Adaptive	3484 (61.19)	0.182	0.168	0.191	−0.313	0.313	−0.533
Depleted–Disconnected	237 (4.16)	−1.001	−0.022	−1.804	0.484	0.167	−0.114
Struggling	1105 (19.41)	−0.467	−0.621	−0.396	0.399	−0.405	0.737
Unfulfilled	358 (6.29)	0.225	0.214	0.356	−0.841	−2.618	−0.395
Engaged–Strained	510 (8.96)	0.193	0.157	0.275	1.501	0.570	2.172

Note. Means are latent class model estimates for standardized indicators. Class sizes and percentages are based on modal assignment.

**Table 5:** Logistic regression results evaluating the effects of demographic characteristics on latent profile membership.

Profile Comparison	Female	Teaching Experience	Class Size	Junior High	Senior High	Suburban	Rural
Broadly Adaptive vs. Depleted–Disconnected	0.76 [0.54, 1.07]	1.12 [0.96, 1.31]	0.90 [0.74, 1.10]	1.04 [0.71, 1.52]	0.89 [0.61, 1.31]	1.10 [0.79, 1.53]	2.44 [1.16, 5.13]
Broadly Adaptive vs. Struggling	1.10 [0.91, 1.34]	**1.30 [1.17, 1.44]**	1.04 [0.94, 1.15]	0.82 [0.65, 1.03]	0.88 [0.68, 1.12]	0.87 [0.71, 1.08]	0.93 [0.67, 1.29]
Broadly Adaptive vs. Unfulfilled	1.22 [0.95, 1.55]	1.12 [1.00, 1.25]	1.08 [0.96, 1.21]	0.95 [0.72, 1.26]	1.17 [0.86, 1.61]	0.96 [0.74, 1.25]	1.33 [0.87, 2.04]
Broadly Adaptive vs. Engaged–Strained	1.18 [0.96, 1.46]	1.10 [0.99, 1.22]	0.92 [0.83, 1.03]	0.79 [0.61, 1.01]	0.85 [0.65, 1.13]	0.74 [0.58, 0.93]	0.62 [0.43, 0.88]
Struggling vs. Depleted–Disconnected	0.69 [0.48, 1.00]	0.86 [0.73, 1.03]	0.87 [0.70, 1.07]	1.27 [0.84, 1.92]	1.02 [0.67, 1.56]	1.26 [0.87, 1.81]	2.62 [1.20, 5.73]
Unfulfilled vs. Depleted–Disconnected	0.63 [0.42, 0.94]	1.00 [0.84, 1.20]	0.83 [0.67, 1.04]	1.10 [0.70, 1.72]	0.76 [0.48, 1.22]	1.14 [0.77, 1.70]	1.83 [0.80, 4.21]
Engaged–Strained vs. Depleted–Disconnected	0.65 [0.44, 0.95]	1.02 [0.85, 1.22]	0.97 [0.79, 1.21]	1.32 [0.86, 2.04]	1.05 [0.67, 1.64]	1.49 [1.01, 2.20]	**3.96 [1.78, 8.80]**
Unfulfilled vs. Struggling	0.91 [0.69, 1.20]	1.16 [1.01, 1.33]	0.96 [0.84, 1.11]	0.86 [0.62, 1.20]	0.75 [0.52, 1.07]	0.91 [0.67, 1.23]	0.70 [0.43, 1.14]
Engaged–Strained vs. Struggling	0.94 [0.71, 1.22]	1.18 [1.03, 1.35]	1.13 [0.98, 1.29]	1.04 [0.76, 1.44]	1.03 [0.72, 1.46]	1.19 [0.88, 1.60]	1.51 [0.96, 2.37]
Engaged–Strained vs. Unfulfilled	1.03 [0.76, 1.39]	1.02 [0.88, 1.17]	1.17 [1.01, 1.36]	1.21 [0.85, 1.73]	1.37 [0.93, 2.04]	1.31 [0.94, 1.82]	2.16 [1.28, 3.65]

Note. *N* = 5694. Each row gives the odds of membership in the first named profile relative to the second named profile. Values are odds ratios with 95% confidence intervals. Female is compared with male, junior high and senior high are compared with primary school, and suburban and rural locations are compared with urban locations. Continuous predictors are standardized. FDR correction was conducted separately within each model across 70 unique profile pair by predictor tests. Bold values indicate Benjamini and Hochberg false discovery rate adjusted *q* < 0.05.

## Data Availability

The raw data supporting the conclusions of this article will be made available by the authors on request.

## Supplementary Materials

The following supporting information can be downloaded at: https://www.mdpi.com/article/10.3390/bs16101850/s1, Table S1. Sample selection and demographic characteristics; Table S2. Measurement scoring, reliability, and confirmatory factor analyses; Table S3. Fit indices and admissibility across six LPA specifications; Table S4. Numerical replication of the S3 solutions; Table S5. Class correspondence across the four-, five-, and six-profile solutions; Table S6. Posterior classification probabilities for the retained solution; Table S7. Complete demographic associations with profile membership; Table S8. Summary of PA item deletion sensitivity analyses; Table S9. Profile sizes following PA item deletion; Table S10. Model-estimated indicator means following PA item deletion; Table S11. Profile retention and numerical diagnostics for PA item deletion; Figure S1. Information criteria across S3 profile solutions; Figure S2. Profile configurations following PA item deletion.
