# Supplementary material for: Beyond a Single Continuum: Latent Profiles of Flourishing and Burnout Among Chinese Teachers
Source: Behav Sci (Basel). 2026 Oct 9;16(10):1850. doi: 10.3390/bs16101850 (PMC13701642; doi:10.3390/bs16101850)
Supplement: Supplementary file 1 [file behavsci-16-01850-s001.zip › behavsci-4522832-supplementary.pdf]

Supplementary Materials

Beyond a Single Continuum: Latent Profiles of Flourishing and Burnout Among Chinese Teachers

Table S1. Sample selection and demographic characteristics

Table S2. Measurement scoring reliability and confirmatory factor analyses

Table S3. Fit indices and admissibility across six LPA specifications

Table S4. Numerical replication of the S3 solutions

Table S5. Class correspondence across the four five and six profile solutions

Table S6. Posterior classification probabilities for the retained solution

Table S7. Complete demographic associations with profile membership

Table S8. Summary of PA item deletion sensitivity analyses

Table S9. Profile sizes following PA item deletion

Table S10. Model estimated indicator means following PA item deletion

Table S11. Profile retention and numerical diagnostics for PA item deletion

Figure S1. Information criteria across S3 profile solutions

Figure S2. Profile configurations following PA item deletion

Table S1 Sample selection and demographic characteristics

A Sample selection

Stage	n
Submitted questionnaires	5,849
Records removed after retaining one record in each confirmed duplicate set	152
Additional records excluded for implausible age and teaching experience combinations	3
Final analytic sample	5,694

Note. The 278 records in 126 confirmed duplicate sets yielded 152 removals.

B Categorical characteristics

Characteristic	Category	n	%
Gender	Male	2,053	36.06
Gender	Female	3,641	63.94
Teaching level	Primary	2,205	38.72
Teaching level	Junior high	1,927	33.84
Teaching level	Senior high	1,562	27.43
School location	Urban	1,719	30.19
School location	Suburban	3,245	56.99
School location	Rural	730	12.82

C Continuous characteristics

Characteristic	M	SD	Observed range
Age in years	37.01	9.55	21 to 65
Teaching experience in years	14.07	10.08	1 to 45
Class size in students	46.52	9.22	4 to 100

Note. N = 5,694 for all characteristics shown. All 32 administered scale items and the demographic variables included in the regression models were complete.

Table S2 Measurement scoring reliability and confirmatory factor analyses

A Item allocation scoring and reliability

Scale	Item codes	Items	α	CR	AVE
EW	F1 F2 F3	3	0.833	0.899	0.747
PW	F4 F5 F6 F7 F8 F9 F10 F11 F12	9	0.903	0.936	0.619
SW	F13 F14 F15 F16 F17	5	0.857	0.903	0.650
EE	B1 B2 B3 B5 B6	5	0.887	0.925	0.713
PA	B4 B7 B10 B12 B13 B14	6	0.860	0.893	0.585
DP	B8 B9 B11 B15	4	0.866	0.907	0.710

Note. All items were scored from 1 to 5. Each composite is an unweighted sum without reverse scoring. Higher PA denotes greater personal accomplishment. The PA items were B4, B7, B10, B12, B13, and B14. The intended item “I feel very energetic” was not administered. CR = composite reliability; AVE = average variance extracted. CR and AVE were calculated from standardized CFA loadings.

B Fit of the three factor measurement models

Fit index	Flourishing	Burnout
χ^2	3960.48	6849.12
df	116	87
CFI	0.970	0.955
TLI	0.965	0.946
RMSEA	0.076	0.117
SRMR	0.031	0.073
RMSEA 90% CI	[0.074, 0.078]	[0.115, 0.119]

Note. N = 5,694. Both models used WLSMV estimation, theta parameterization, and ordered categorical indicators. Both model χ^2 tests had $p < .001$. The full set of fit indices is reported because the indices did not uniformly indicate close fit, particularly for the burnout model.

Table S2 Continued

C Standardized factor loadings

Measure	Factor	Item code	Standardized loading
Flourishing	EW	F1	0.870
Flourishing	EW	F2	0.885
Flourishing	EW	F3	0.837
Flourishing	PW	F4	0.786
Flourishing	PW	F5	0.790
Flourishing	PW	F6	0.767
Flourishing	PW	F7	0.808
Flourishing	PW	F8	0.789
Flourishing	PW	F9	0.782
Flourishing	PW	F10	0.766
Flourishing	PW	F11	0.822
Flourishing	PW	F12	0.770
Flourishing	SW	F13	0.826
Flourishing	SW	F14	0.843
Flourishing	SW	F15	0.795
Flourishing	SW	F16	0.779
Flourishing	SW	F17	0.786
Burnout	EE	B1	0.825
Burnout	EE	B2	0.895
Burnout	EE	B3	0.845
Burnout	EE	B5	0.818
Burnout	EE	B6	0.836
Burnout	PA	B4	0.637
Burnout	PA	B7	0.675
Burnout	PA	B10	0.739
Burnout	PA	B12	0.857
Burnout	PA	B13	0.867
Burnout	PA	B14	0.784
Burnout	DP	B8	0.915
Burnout	DP	B9	0.888
Burnout	DP	B11	0.793
Burnout	DP	B15	0.766

Note. Loadings are fully standardized estimates from the ordered categorical measurement models.

Table S3 Fit indices and admissibility across six LPA specifications

S1 Equal variances, all within-profile covariances fixed to zero.

K	Loglikelihood	AIC	BIC	aBIC	Entropy	LMR p	Status
1	-48473.616	96971.232	97050.998	97012.865	NA	NA	Admissible
2	-45548.356	91134.711	91261.008	91200.631	0.762	< .001	Admissible
3	-43396.459	86844.919	87017.745	86935.125	0.874	< .001	Admissible
4	-41723.582	83513.164	83732.520	83627.656	0.885	< .001	Admissible
5	-40685.910	81451.820	81717.706	81590.598	0.900	< .001	Admissible
6	-40019.319	80132.638	80445.055	80295.703	0.901	0.002	Admissible
7	-39518.061	79144.122	79503.069	79331.473	0.895	0.008	Admissible

S2 Profile-specific variances, all covariances fixed to zero.

K	Loglikelihood	AIC	BIC	aBIC	Entropy	LMR p	Status
1	-48473.616	96971.232	97050.998	97012.865	NA	NA	Admissible
2	-44567.347	89184.694	89350.873	89271.431	0.862	< .001	Admissible
3	NA	NA	NA	NA	NA	NA	Nonconvergent
4	NA	NA	NA	NA	NA	NA	Nonconvergent
5	NA	NA	NA	NA	NA	NA	Nonconvergent
6	NA	NA	NA	NA	NA	NA	Nonconvergent
7	NA	NA	NA	NA	NA	NA	Nonconvergent

Note. N = 5,694. Search settings were 2,000 initial and 500 final starts, with 100 initial iterations. NA denotes an unavailable or inapplicable estimate. Nonconvergent solutions are not valid model estimates. S2 showed negative variance diagnostics from K3 onward. Corrected S4 and S6 outputs implement profile-specific covariance parameters. Their higher-class runs did not terminate normally under these search settings. The targeted covariances in S5 and S6 are EW with PW, EW with SW, PW with SW, and EE with DP. LMR p is the adjusted Lo-Mendell-Rubin test. AIC = Akaike information criterion; BIC = Bayesian information criterion; aBIC = sample-size-adjusted BIC.

Table S3 Continued

S3 Equal variances, all 15 covariances freely estimated and equal across profiles.

K	Loglikelihood	AIC	BIC	aBIC	Entropy	LMR p	Status
1	-40997.389	82048.779	82228.252	82142.454	NA	NA	Admissible
2	-39837.015	79742.031	79968.035	79859.993	0.972	< .001	Admissible
3	-38888.741	77859.481	78132.015	78001.729	0.944	< .001	Admissible
4	-38346.389	76788.778	77107.842	76955.312	0.928	< .001	Admissible
5	-38105.643	76321.286	76686.881	76512.107	0.838	< .001	Admissible
6	-37855.704	75835.407	76247.532	76050.514	0.859	< .001	Admissible
7	-37641.693	75421.387	75880.041	75660.780	0.871	0.226	Admissible

S4 Profile-specific variances and all 15 covariances freely estimated by profile.

K	Loglikelihood	AIC	BIC	aBIC	Entropy	LMR p	Status
1	-40997.389	82048.779	82228.252	82142.454	NA	NA	Admissible
2	-38143.073	76396.146	76761.740	76586.967	0.867	< .001	Admissible
3	NA	NA	NA	NA	NA	NA	Nonconvergent
4	NA	NA	NA	NA	NA	NA	Nonconvergent
5	NA	NA	NA	NA	NA	NA	Nonconvergent
6	NA	NA	NA	NA	NA	NA	Nonconvergent
7	NA	NA	NA	NA	NA	NA	Nonconvergent

Note. N = 5,694. Search settings were 2,000 initial and 500 final starts, with 100 initial iterations. NA denotes an unavailable or inapplicable estimate. Nonconvergent solutions are not valid model estimates. S2 showed negative variance diagnostics from K3 onward. Corrected S4 and S6 outputs implement profile-specific covariance parameters. Their higher-class runs did not terminate normally under these search settings. The targeted covariances in S5 and S6 are EW with PW, EW with SW, PW with SW, and EE with DP. LMR p is the adjusted Lo-Mendell-Rubin test. AIC = Akaike information criterion; BIC = Bayesian information criterion; aBIC = sample-size-adjusted BIC.

Table S3 Continued

S5 Equal variances and four targeted covariances equal across profiles.

K	Loglikelihood	AIC	BIC	aBIC	Entropy	LMR p	Status
1	-41936.751	83905.503	84011.857	83961.014	NA	NA	Admissible
2	-41145.481	82336.962	82489.847	82416.760	0.948	< .001	Admissible
3	-40316.969	80693.939	80893.354	80798.023	0.869	< .001	Admissible
4	-39328.344	78730.689	78976.634	78859.059	0.810	< .001	Admissible
5	-38774.966	77637.933	77930.408	77790.589	0.816	< .001	Admissible
6	-38304.960	76711.920	77050.925	76888.863	0.824	< .001	Admissible
7	-38053.915	76223.831	76609.367	76425.060	0.831	0.150	Admissible

S6 Profile-specific variances and four targeted covariances freely estimated by profile.

K	Loglikelihood	AIC	BIC	aBIC	Entropy	LMR p	Status
1	-41936.751	83905.503	84011.857	83961.014	NA	NA	Admissible
2	NA	NA	NA	NA	NA	NA	Nonconvergent
3	NA	NA	NA	NA	NA	NA	Nonconvergent
4	NA	NA	NA	NA	NA	NA	Nonconvergent
5	NA	NA	NA	NA	NA	NA	Nonconvergent
6	NA	NA	NA	NA	NA	NA	Nonconvergent
7	NA	NA	NA	NA	NA	NA	Nonconvergent

Note. N = 5,694. Search settings were 2,000 initial and 500 final starts, with 100 initial iterations. NA denotes an unavailable or inapplicable estimate. Nonconvergent solutions are not valid model estimates. S2 showed negative variance diagnostics from K3 onward. Corrected S4 and S6 outputs implement profile-specific covariance parameters. Their higher-class runs did not terminate normally under these search settings. The targeted covariances in S5 and S6 are EW with PW, EW with SW, PW with SW, and EE with DP. LMR p is the adjusted Lo-Mendell-Rubin test. AIC = Akaike information criterion; BIC = Bayesian information criterion; aBIC = sample-size-adjusted BIC.

Table S4 Numerical replication of the S3 solutions

A Broad search replication

K	Loglikelihood	Best likelihood occurrences	Normal termination
1	-40997.389	499	Yes
2	-39837.015	387	Yes
3	-38888.741	296	Yes
4	-38346.389	240	Yes
5	-38105.643	291	Yes
6	-37855.704	46	Yes
7	-37641.693	99	Yes

B Confirmation with increased random starts

K	Broad search loglikelihood	Confirmation loglikelihood	Modal agreement	ARI
5	-38105.643	-38105.643	1.000	1.000
6	-37855.704	-37855.704	1.000	1.000
7	-37641.693	-37641.693	1.000	1.000

Note. Broad searches used STARTS = 2000 500 and STITERATIONS = 100. Confirmation runs for K5, K6, and K7 used STARTS = 5000 1000. Modal agreement is a proportion after class label alignment. The best loglikelihood and modal classifications were reproduced in each confirmation. ARI = adjusted Rand index. A one-profile model has no classification entropy or likelihood ratio test against a zero-profile model.

Table S5 Class correspondence across the four five and six profile solutions

A K4 to K5 modal classification correspondence

K4 profile	Broadly Adaptive	Depleted-Disconnected	Struggling	Unfulfilled	Engaged-Strained	Total
Unfulfilled	1 (0.29%)	0 (0.00%)	0 (0.00%)	339 (99.71%)	0 (0.00%)	340
Broad central	3463 (77.32%)	1 (0.02%)	996 (22.24%)	19 (0.42%)	0 (0.00%)	4479
Engaged-Strained	0 (0.00%)	0 (0.00%)	93 (15.47%)	0 (0.00%)	508 (84.53%)	601
Depleted-Disconnected	20 (7.30%)	236 (86.13%)	16 (5.84%)	0 (0.00%)	2 (0.73%)	274

B K5 to K6 modal classification correspondence

K5 profile	Extreme Unfulfilled	Moderate Unfulfilled	Struggling	Depleted-Disconnected	Broadly Adaptive	Engaged-Strained	Total
Broadly Adaptive	0 (0.00%)	46 (1.32%)	2 (0.06%)	5 (0.14%)	3431 (98.48%)	0 (0.00%)	3484
Depleted-Disconnected	0 (0.00%)	0 (0.00%)	0 (0.00%)	230 (97.05%)	7 (2.95%)	0 (0.00%)	237
Struggling	0 (0.00%)	60 (5.43%)	900 (81.45%)	1 (0.09%)	143 (12.94%)	1 (0.09%)	1105
Unfulfilled	122 (34.08%)	236 (65.92%)	0 (0.00%)	0 (0.00%)	0 (0.00%)	0 (0.00%)	358
Engaged-Strained	0 (0.00%)	0 (0.00%)	22 (4.31%)	0 (0.00%)	0 (0.00%)	488 (95.69%)	510

Note. Cells give n (percentage of the row profile). These are assignments of the same teachers under different class counts, not longitudinal transitions. K4 Broad central contributed 3,463 teachers to K5 Broadly Adaptive and 996 to K5 Struggling. The latter accounted for 90.14% of K5 Struggling. Of the 358 K5 Unfulfilled teachers, 236 entered Moderate Unfulfilled and 122 entered Extreme Unfulfilled at K6.

C Similarity of the two K6 descendants to K5 Unfulfilled

K6 descendant	Total n (%)	From K5 Unfulfilled n (%)	Shape correlation
Extreme Unfulfilled	122 (2.14%)	122 (34.08%)	0.990
Moderate Unfulfilled	342 (6.01%)	236 (65.92%)	0.988

Note. Shape correlations compare the six model-estimated standardized indicator means. The percentage in the third column uses K5 Unfulfilled, n = 358, as its denominator.

Table S6 Posterior classification probabilities for the retained solution

A Modal and model expected class sizes

Profile	Modal n (%)	Expected n (%)	Mean assigned probability
Broadly Adaptive	3,484 (61.19%)	3402.20 (59.75%)	0.917
Depleted-Disconnected	237 (4.16%)	252.88 (4.44%)	0.872
Struggling	1,105 (19.41%)	1166.17 (20.48%)	0.791
Unfulfilled	358 (6.29%)	354.92 (6.23%)	0.942
Engaged-Strained	510 (8.96%)	517.50 (9.09%)	0.931

B Average posterior probability matrix

Modal profile	BA	DD	ST	UF	ES
Broadly Adaptive	0.917	0.011	0.069	0.003	0.000
Depleted-Disconnected	0.090	0.872	0.037	0.000	0.001
Struggling	0.157	0.007	0.791	0.007	0.038
Unfulfilled	0.031	0.001	0.027	0.942	0.000
Engaged-Strained	0.003	0.001	0.065	0.000	0.931

Note. Rows refer to modal assignments and columns to posterior membership probabilities. Model expected sizes are sums of posterior probabilities and need not equal modal counts. Overall entropy was .838. The mean assigned probability was .791 for Struggling, indicating greater classification uncertainty than in the other profiles. Probabilities derived from rounded posterior outputs may not sum to exactly 1.000. BA = Broadly Adaptive; DD = Depleted-Disconnected; ST = Struggling; UF = Unfulfilled; ES = Engaged-Strained.

Table S7 Teaching experience model

Profile contrast	Predictor	B	SE	OR	95% CI	p	q
BA vs DD	Female	-0.269	0.174	0.764	[0.543, 1.075]	0.122	0.305
	Teaching experience	0.117	0.080	1.124	[0.961, 1.315]	0.142	0.334
	Class size	-0.106	0.101	0.899	[0.738, 1.096]	0.293	0.460
	Junior high	0.040	0.193	1.041	[0.713, 1.519]	0.838	0.889
	Senior high	-0.111	0.196	0.895	[0.609, 1.314]	0.571	0.746
	Suburban	0.092	0.169	1.096	[0.787, 1.527]	0.586	0.746
	Rural	0.891	0.380	2.438	[1.157, 5.134]	0.019	0.166
BA vs ST	Female	0.099	0.100	1.104	[0.908, 1.343]	0.322	0.479
	Teaching experience	0.263	0.052	1.301	[1.175, 1.440]	< .001	< .001
	Class size	0.037	0.051	1.038	[0.939, 1.147]	0.460	0.657
	Junior high	-0.199	0.117	0.820	[0.652, 1.031]	0.087	0.305
	Senior high	-0.132	0.127	0.876	[0.683, 1.124]	0.295	0.460
	Suburban	-0.137	0.108	0.872	[0.706, 1.078]	0.204	0.392
	Rural	-0.073	0.166	0.930	[0.671, 1.287]	0.658	0.809
BA vs UF	Female	0.196	0.124	1.217	[0.954, 1.551]	0.115	0.305
	Teaching experience	0.113	0.057	1.120	[1.001, 1.252]	0.048	0.230
	Class size	0.076	0.060	1.079	[0.959, 1.214]	0.207	0.392
	Junior high	-0.051	0.144	0.950	[0.717, 1.260]	0.722	0.856
	Senior high	0.160	0.161	1.174	[0.856, 1.609]	0.320	0.479
	Suburban	-0.040	0.133	0.961	[0.740, 1.247]	0.764	0.889
	Rural	0.287	0.218	1.332	[0.869, 2.043]	0.189	0.389

Note. The first profile is compared with the second. Female is relative to male, teaching levels to primary school, and locations to urban school location. Continuous predictors are standardized (SD: age 9.546 years, teaching experience 10.078 years, class size 9.216 students). Age and teaching experience were entered in separate models (r = .959). B = log odds coefficient. p values were reconstructed from printed Mplus z statistics. Benjamini and Hochberg q values correct 70 unique contrasts within each model. BA = Broadly Adaptive; DD = Depleted-Disconnected; ST = Struggling; UF = Unfulfilled; ES = Engaged-Strained.

Table S7 Teaching experience model continued

Profile contrast	Predictor	B	SE	OR	95% CI	p	q
BA vs ES	Female	0.167	0.108	1.182	[0.956, 1.460]	0.122	0.305
	Teaching experience	0.097	0.053	1.102	[0.993, 1.222]	0.067	0.275
	Class size	-0.081	0.055	0.922	[0.828, 1.027]	0.143	0.334
	Junior high	-0.242	0.129	0.785	[0.610, 1.011]	0.062	0.270
	Senior high	-0.158	0.142	0.854	[0.646, 1.128]	0.267	0.446
	Suburban	-0.307	0.122	0.736	[0.579, 0.934]	0.012	0.166
	Rural	-0.484	0.181	0.616	[0.432, 0.879]	0.007	0.129
ST vs DD	Female	-0.368	0.187	0.692	[0.480, 0.999]	0.049	0.230
	Teaching experience	-0.146	0.089	0.864	[0.726, 1.029]	0.101	0.305
	Class size	-0.144	0.108	0.866	[0.701, 1.070]	0.184	0.389
	Junior high	0.239	0.210	1.270	[0.841, 1.917]	0.256	0.446
	Senior high	0.021	0.216	1.021	[0.669, 1.559]	0.923	0.936
	Suburban	0.229	0.186	1.257	[0.873, 1.810]	0.218	0.402
	Rural	0.964	0.399	2.622	[1.200, 5.732]	0.016	0.166
UF vs DD	Female	-0.465	0.203	0.628	[0.422, 0.935]	0.022	0.173
	Teaching experience	0.004	0.093	1.004	[0.837, 1.205]	0.970	0.970
	Class size	-0.182	0.113	0.834	[0.668, 1.040]	0.107	0.305
	Junior high	0.091	0.229	1.095	[0.699, 1.716]	0.691	0.834
	Senior high	-0.272	0.241	0.762	[0.475, 1.222]	0.259	0.446
	Suburban	0.132	0.204	1.141	[0.765, 1.702]	0.518	0.710
	Rural	0.604	0.425	1.829	[0.795, 4.208]	0.155	0.338

Note. The first profile is compared with the second. Female is relative to male, teaching levels to primary school, and locations to urban school location. Continuous predictors are standardized (SD: age 9.546 years, teaching experience 10.078 years, class size 9.216 students). Age and teaching experience were entered in separate models (r = .959). B = log odds coefficient. p values were reconstructed from printed Mplus z statistics. Benjamini and Hochberg q values correct 70 unique contrasts within each model. BA = Broadly Adaptive; DD = Depleted-Disconnected; ST = Struggling; UF = Unfulfilled; ES = Engaged-Strained.

Table S7 Teaching experience model continued

Profile contrast	Predictor	B	SE	OR	95% CI	p	q
ES vs DD	Female	-0.436	0.195	0.647	[0.441, 0.948]	0.025	0.177
	Teaching experience	0.020	0.091	1.020	[0.854, 1.219]	0.824	0.889
	Class size	-0.026	0.110	0.974	[0.785, 1.209]	0.817	0.889
	Junior high	0.281	0.221	1.324	[0.859, 2.042]	0.203	0.392
	Senior high	0.046	0.230	1.047	[0.667, 1.643]	0.841	0.889
	Suburban	0.399	0.198	1.490	[1.011, 2.197]	0.044	0.230
	Rural	1.375	0.408	3.955	[1.778, 8.799]	< .001	0.026
UF vs ST	Female	-0.096	0.144	0.908	[0.685, 1.205]	0.504	0.706
	Teaching experience	0.149	0.070	1.161	[1.012, 1.331]	0.034	0.213
	Class size	-0.039	0.071	0.962	[0.837, 1.105]	0.586	0.746
	Junior high	-0.148	0.168	0.862	[0.620, 1.199]	0.380	0.554
	Senior high	-0.293	0.186	0.746	[0.518, 1.074]	0.117	0.305
	Suburban	-0.097	0.156	0.908	[0.668, 1.232]	0.535	0.721
	Rural	-0.360	0.252	0.698	[0.426, 1.143]	0.153	0.338
ES vs ST	Female	-0.067	0.137	0.935	[0.715, 1.223]	0.623	0.778
	Teaching experience	0.166	0.070	1.181	[1.029, 1.354]	0.018	0.166
	Class size	0.118	0.071	1.125	[0.979, 1.293]	0.096	0.305
	Junior high	0.043	0.164	1.044	[0.757, 1.440]	0.795	0.889
	Senior high	0.025	0.179	1.025	[0.722, 1.456]	0.888	0.914
	Suburban	0.170	0.154	1.185	[0.876, 1.603]	0.267	0.446
	Rural	0.411	0.230	1.508	[0.961, 2.367]	0.073	0.286

Note. The first profile is compared with the second. Female is relative to male, teaching levels to primary school, and locations to urban school location. Continuous predictors are standardized (SD: age 9.546 years, teaching experience 10.078 years, class size 9.216 students). Age and teaching experience were entered in separate models ($r = .959$). B = log odds coefficient. p values were reconstructed from printed Mplus z statistics. Benjamini and Hochberg q values correct 70 unique contrasts within each model. BA = Broadly Adaptive; DD = Depleted-Disconnected; ST = Struggling; UF = Unfulfilled; ES = Engaged-Strained.

Table S7 Teaching experience model continued

Profile contrast	Predictor	B	SE	OR	95% CI	p	q
ES vs UF	Female	0.029	0.154	1.029	[0.761, 1.392]	0.851	0.889
	Teaching experience	0.017	0.073	1.017	[0.882, 1.174]	0.820	0.889
	Class size	0.157	0.077	1.170	[1.006, 1.361]	0.042	0.230
	Junior high	0.190	0.182	1.209	[0.846, 1.728]	0.296	0.460
	Senior high	0.318	0.202	1.374	[0.925, 2.042]	0.116	0.305
	Suburban	0.267	0.170	1.306	[0.936, 1.822]	0.117	0.305
	Rural	0.771	0.267	2.162	[1.281, 3.649]	0.004	0.092

Note. The first profile is compared with the second. Female is relative to male, teaching levels to primary school, and locations to urban school location. Continuous predictors are standardized (SD: age 9.546 years, teaching experience 10.078 years, class size 9.216 students). Age and teaching experience were entered in separate models (r = .959). B = log odds coefficient. p values were reconstructed from printed Mplus z statistics. Benjamini and Hochberg q values correct 70 unique contrasts within each model. BA = Broadly Adaptive; DD = Depleted-Disconnected; ST = Struggling; UF = Unfulfilled; ES = Engaged-Strained.

Table S7 Age model

Profile contrast	Predictor	B	SE	OR	95% CI	p	q
BA vs DD	Female	-0.272	0.174	0.762	[0.542, 1.071]	0.119	0.329
	Age	0.094	0.079	1.099	[0.941, 1.283]	0.237	0.426
	Class size	-0.105	0.101	0.900	[0.739, 1.097]	0.298	0.471
	Junior high	0.036	0.193	1.037	[0.710, 1.513]	0.854	0.887
	Senior high	-0.118	0.196	0.889	[0.605, 1.305]	0.549	0.724
	Suburban	0.093	0.169	1.097	[0.788, 1.528]	0.581	0.740
	Rural	0.894	0.380	2.445	[1.161, 5.149]	0.019	0.164
BA vs ST	Female	0.099	0.100	1.104	[0.908, 1.343]	0.321	0.489
	Age	0.258	0.051	1.294	[1.171, 1.430]	< .001	< .001
	Class size	0.039	0.050	1.040	[0.943, 1.147]	0.442	0.631
	Junior high	-0.207	0.117	0.813	[0.646, 1.023]	0.076	0.281
	Senior high	-0.151	0.126	0.860	[0.672, 1.101]	0.233	0.426
	Suburban	-0.133	0.108	0.875	[0.708, 1.082]	0.218	0.424
	Rural	-0.074	0.166	0.929	[0.671, 1.286]	0.656	0.792
BA vs UF	Female	0.198	0.124	1.219	[0.956, 1.554]	0.112	0.329
	Age	0.119	0.058	1.126	[1.005, 1.262]	0.041	0.225
	Class size	0.076	0.060	1.079	[0.959, 1.214]	0.206	0.412
	Junior high	-0.056	0.145	0.946	[0.712, 1.256]	0.699	0.815
	Senior high	0.151	0.161	1.163	[0.848, 1.594]	0.350	0.521
	Suburban	-0.038	0.133	0.963	[0.742, 1.249]	0.776	0.887
	Rural	0.286	0.218	1.331	[0.868, 2.041]	0.190	0.403

Note. The first profile is compared with the second. Female is relative to male, teaching levels to primary school, and locations to urban school location. Continuous predictors are standardized (SD: age 9.546 years, teaching experience 10.078 years, class size 9.216 students). Age and teaching experience were entered in separate models (r = .959). B = log odds coefficient. p values were reconstructed from printed Mplus z statistics. Benjamini and Hochberg q values correct 70 unique contrasts within each model. BA = Broadly Adaptive; DD = Depleted-Disconnected; ST = Struggling; UF = Unfulfilled; ES = Engaged-Strained.

Table S7 Age model continued

Profile contrast	Predictor	B	SE	OR	95% CI	p	q
BA vs ES	Female	0.164	0.108	1.178	[0.953, 1.456]	0.128	0.331
	Age	0.078	0.053	1.081	[0.974, 1.199]	0.141	0.345
	Class size	-0.080	0.055	0.923	[0.829, 1.028]	0.147	0.345
	Junior high	-0.244	0.130	0.783	[0.607, 1.011]	0.060	0.259
	Senior high	-0.162	0.142	0.850	[0.644, 1.123]	0.255	0.431
	Suburban	-0.306	0.122	0.736	[0.580, 0.935]	0.013	0.146
	Rural	-0.483	0.180	0.617	[0.434, 0.878]	0.008	0.132
ST vs DD	Female	-0.372	0.188	0.689	[0.477, 0.996]	0.048	0.225
	Age	-0.165	0.088	0.848	[0.714, 1.008]	0.063	0.259
	Class size	-0.144	0.108	0.866	[0.701, 1.070]	0.183	0.399
	Junior high	0.242	0.210	1.274	[0.844, 1.922]	0.248	0.431
	Senior high	0.033	0.216	1.034	[0.677, 1.578]	0.879	0.892
	Suburban	0.226	0.186	1.254	[0.871, 1.805]	0.225	0.425
	Rural	0.967	0.399	2.630	[1.203, 5.749]	0.015	0.155
UF vs DD	Female	-0.470	0.204	0.625	[0.419, 0.932]	0.021	0.164
	Age	-0.025	0.094	0.975	[0.811, 1.173]	0.789	0.887
	Class size	-0.181	0.113	0.834	[0.669, 1.041]	0.109	0.329
	Junior high	0.092	0.229	1.096	[0.700, 1.717]	0.689	0.815
	Senior high	-0.268	0.241	0.765	[0.477, 1.227]	0.265	0.431
	Suburban	0.131	0.204	1.140	[0.764, 1.700]	0.520	0.714
	Rural	0.608	0.425	1.837	[0.799, 4.225]	0.153	0.345

Note. The first profile is compared with the second. Female is relative to male, teaching levels to primary school, and locations to urban school location. Continuous predictors are standardized (SD: age 9.546 years, teaching experience 10.078 years, class size 9.216 students). Age and teaching experience were entered in separate models ($r = .959$). B = log odds coefficient. p values were reconstructed from printed Mplus z statistics. Benjamini and Hochberg q values correct 70 unique contrasts within each model. BA = Broadly Adaptive; DD = Depleted-Disconnected; ST = Struggling; UF = Unfulfilled; ES = Engaged-Strained.

Table S7 Age model continued

Profile contrast	Predictor	B	SE	OR	95% CI	p	q
ES vs DD	Female	-0.436	0.195	0.647	[0.441, 0.948]	0.025	0.178
	Age	0.016	0.091	1.016	[0.850, 1.215]	0.862	0.887
	Class size	-0.025	0.110	0.975	[0.786, 1.210]	0.820	0.887
	Junior high	0.279	0.221	1.322	[0.857, 2.038]	0.206	0.412
	Senior high	0.044	0.230	1.045	[0.666, 1.640]	0.847	0.887
	Suburban	0.399	0.198	1.490	[1.011, 2.197]	0.044	0.225
	Rural	1.376	0.408	3.959	[1.779, 8.808]	< .001	0.026
UF vs ST	Female	-0.098	0.144	0.907	[0.684, 1.202]	0.495	0.693
	Age	0.140	0.071	1.150	[1.001, 1.322]	0.048	0.225
	Class size	-0.038	0.071	0.963	[0.838, 1.106]	0.599	0.749
	Junior high	-0.151	0.168	0.860	[0.619, 1.195]	0.370	0.539
	Senior high	-0.301	0.187	0.740	[0.513, 1.068]	0.106	0.329
	Suburban	-0.095	0.156	0.909	[0.670, 1.235]	0.545	0.724
	Rural	-0.360	0.252	0.698	[0.426, 1.143]	0.153	0.345
ES vs ST	Female	-0.065	0.137	0.937	[0.716, 1.226]	0.637	0.782
	Age	0.180	0.070	1.197	[1.044, 1.373]	0.010	0.138
	Class size	0.119	0.071	1.126	[0.980, 1.295]	0.094	0.328
	Junior high	0.037	0.164	1.038	[0.752, 1.431]	0.821	0.887
	Senior high	0.011	0.179	1.011	[0.712, 1.436]	0.950	0.950
	Suburban	0.173	0.154	1.189	[0.879, 1.608]	0.261	0.431
	Rural	0.409	0.229	1.505	[0.961, 2.358]	0.074	0.281

Note. The first profile is compared with the second. Female is relative to male, teaching levels to primary school, and locations to urban school location. Continuous predictors are standardized (SD: age 9.546 years, teaching experience 10.078 years, class size 9.216 students). Age and teaching experience were entered in separate models (r = .959). B = log odds coefficient. p values were reconstructed from printed Mplus z statistics. Benjamini and Hochberg q values correct 70 unique contrasts within each model. BA = Broadly Adaptive; DD = Depleted-Disconnected; ST = Struggling; UF = Unfulfilled; ES = Engaged-Strained.

Table S7 Age model continued

Profile contrast	Predictor	B	SE	OR	95% CI	p	q
ES vs UF	Female	0.034	0.154	1.035	[0.765, 1.399]	0.827	0.887
	Age	0.041	0.074	1.042	[0.901, 1.204]	0.582	0.740
	Class size	0.156	0.077	1.169	[1.005, 1.359]	0.043	0.225
	Junior high	0.188	0.182	1.207	[0.845, 1.724]	0.303	0.471
	Senior high	0.313	0.202	1.368	[0.920, 2.032]	0.122	0.329
	Suburban	0.268	0.171	1.307	[0.935, 1.828]	0.116	0.329
	Rural	0.768	0.267	2.155	[1.277, 3.638]	0.004	0.094

Note. The first profile is compared with the second. Female is relative to male, teaching levels to primary school, and locations to urban school location. Continuous predictors are standardized (SD: age 9.546 years, teaching experience 10.078 years, class size 9.216 students). Age and teaching experience were entered in separate models (r = .959). B = log odds coefficient. p values were reconstructed from printed Mplus z statistics. Benjamini and Hochberg q values correct 70 unique contrasts within each model. BA = Broadly Adaptive; DD = Depleted-Disconnected; ST = Struggling; UF = Unfulfilled; ES = Engaged-Strained.

Table S8 Summary of PA item deletion sensitivity analyses

One of the six administered PA items was omitted in each analysis. PA was recalculated as the sum of the remaining five items, and all six composites were restandardized in the same sample. Each model retained the primary S3 specification and five profiles. Profiles were aligned to the baseline solution before comparison.

Omitted item	Entropy	Agreement %	ARI	Maximum mean shift	Largest size change pp	All configurations recovered
B4	0.839	98.19	0.957	0.120	0.53	Yes
B7	0.838	97.70	0.941	0.104	1.00	Yes
B10	0.835	98.14	0.957	0.045	0.39	Yes
B12	0.837	97.44	0.933	0.083	1.11	Yes
B13	0.919	76.24	0.546	1.325	14.23	No
B14	0.835	98.35	0.964	0.138	0.63	Yes

Note. Agreement refers to modal classifications. Mean shifts are absolute differences between standardized model-estimated indicator means. pp = percentage points. B13 was the exception to close recovery: agreement was 76.24%, ARI was .546, and the largest centroid shift was 1.325 SD. Its independently seeded confirmation reproduced the selected likelihood. B13 concerns perceived positive influence on other people's lives through work. These analyses assess sensitivity within the administered PA content and cannot determine the effect of the unadministered item or establish equivalence to the complete scale.

Table S9 Profile sizes following PA item deletion

Omitted item	Broadly Adaptive	Depleted-Disconnected	Struggling	Unfulfilled	Engaged-Strained
Baseline	3484 (61.19%)	237 (4.16%)	1105 (19.41%)	358 (6.29%)	510 (8.96%)
B4	3502 (61.50%)	239 (4.20%)	1117 (19.62%)	328 (5.76%)	508 (8.92%)
B7	3541 (62.19%)	239 (4.20%)	1079 (18.95%)	328 (5.76%)	507 (8.90%)
B10	3494 (61.36%)	240 (4.21%)	1107 (19.44%)	336 (5.90%)	517 (9.08%)
B12	3547 (62.29%)	237 (4.16%)	1052 (18.48%)	361 (6.34%)	497 (8.73%)
B13	4294 (75.41%)	266 (4.67%)	405 (7.11%)	132 (2.32%)	597 (10.48%)
B14	3448 (60.56%)	246 (4.32%)	1103 (19.37%)	392 (6.88%)	505 (8.87%)

Note. Cells give modal n (%), with N = 5,694 in every analysis. Profile names identify the baseline profiles to which the sensitivity classes were matched. Matching labels do not establish substantive recovery, particularly after deleting B13.

Table S10 Model estimated indicator means following PA item deletion

Omitted item	Matched profile	EW	PW	SW	EE	PA	DP
Baseline	Broadly Adaptive	0.182	0.168	0.191	-0.313	0.313	-0.533
Baseline	Depleted-Disconnected	-1.001	-0.022	-1.804	0.484	0.167	-0.114
Baseline	Struggling	-0.467	-0.621	-0.396	0.399	-0.405	0.737
Baseline	Unfulfilled	0.225	0.214	0.356	-0.841	-2.618	-0.395
Baseline	Engaged-Strained	0.193	0.157	0.275	1.501	0.570	2.172
B4	Unfulfilled	0.204	0.186	0.333	-0.801	-2.738	-0.399
B4	Depleted-Disconnected	-1.010	-0.020	-1.801	0.493	0.179	-0.112
B4	Broadly Adaptive	0.185	0.174	0.197	-0.320	0.313	-0.531
B4	Struggling	-0.474	-0.636	-0.409	0.396	-0.412	0.735
B4	Engaged-Strained	0.201	0.167	0.277	1.494	0.515	2.168
B7	Depleted-Disconnected	-1.005	-0.013	-1.803	0.483	0.181	-0.116
B7	Unfulfilled	0.191	0.178	0.323	-0.821	-2.722	-0.388
B7	Broadly Adaptive	0.187	0.176	0.198	-0.316	0.310	-0.530
B7	Engaged-Strained	0.197	0.165	0.284	1.491	0.551	2.162
B7	Struggling	-0.483	-0.645	-0.418	0.398	-0.428	0.736
B10	Broadly Adaptive	0.186	0.174	0.198	-0.319	0.309	-0.534
B10	Unfulfilled	0.226	0.210	0.353	-0.837	-2.663	-0.402
B10	Depleted-Disconnected	-1.000	-0.019	-1.798	0.485	0.145	-0.112
B10	Engaged-Strained	0.183	0.150	0.269	1.493	0.556	2.164
B10	Struggling	-0.470	-0.629	-0.407	0.393	-0.418	0.724

Note. Means are latent class model estimates, not means computed after modal assignment. Sensitivity profile labels indicate alignment with the baseline and do not imply that the original configurations were retained after B13 deletion. EW = emotional well-being; PW = psychological well-being; SW = social well-being; EE = emotional exhaustion; PA = personal accomplishment; DP = depersonalization.

Table S10 Continued

Omitted item	Matched profile	EW	PW	SW	EE	PA	DP
B12	Broadly Adaptive	0.171	0.157	0.181	-0.308	0.302	-0.528
B12	Struggling	-0.461	-0.615	-0.387	0.418	-0.389	0.761
B12	Unfulfilled	0.238	0.221	0.367	-0.850	-2.535	-0.396
B12	Depleted-Disconnected	-0.994	-0.024	-1.807	0.479	0.187	-0.115
B12	Engaged-Strained	0.208	0.178	0.290	1.508	0.589	2.181
B13	Unfulfilled	0.591	0.683	0.791	-1.193	-3.292	-0.741
B13	Depleted-Disconnected	-0.961	-0.075	-1.782	0.492	0.164	-0.053
B13	Struggling	0.073	-0.012	0.168	-0.530	-1.730	-0.081
B13	Broadly Adaptive	0.023	-0.025	0.049	-0.147	0.191	-0.263
B13	Engaged-Strained	0.118	0.067	0.214	1.399	0.532	2.042
B14	Depleted-Disconnected	-0.982	-0.022	-1.796	0.477	0.162	-0.113
B14	Unfulfilled	0.229	0.224	0.358	-0.834	-2.480	-0.381
B14	Struggling	-0.473	-0.629	-0.399	0.406	-0.392	0.737
B14	Engaged-Strained	0.188	0.151	0.277	1.503	0.586	2.173
B14	Broadly Adaptive	0.182	0.169	0.190	-0.310	0.323	-0.534

Note. Means are latent class model estimates, not means computed after modal assignment. Sensitivity profile labels indicate alignment with the baseline and do not imply that the original configurations were retained after B13 deletion. EW = emotional well-being; PW = psychological well-being; SW = social well-being; EE = emotional exhaustion; PA = personal accomplishment; DP = depersonalization.

Table S11 Profile retention and numerical diagnostics for PA item deletion

A Retention of teachers assigned to each baseline profile

Omitted item	BA %	DD %	ST %	UF %	ES %
B4	99.23	99.58	97.74	89.11	97.84
B7	99.40	99.58	94.75	88.27	98.24
B10	99.17	100.00	96.83	90.50	98.43
B12	99.31	99.16	92.31	94.97	96.67
B13	96.99	97.89	8.14	36.87	99.61
B14	98.65	100.00	97.47	97.49	98.04

B Diagnostics of the selected runs

Omitted item	Run	Initial / final starts	Seed	Loglikelihood	Best likelihood occurrences	Normal termination
B4	primary	2000 / 500	260902	-38108.849	188 / 500	Yes
B7	primary	2000 / 500	260902	-38058.959	188 / 500	Yes
B10	primary	2000 / 500	260902	-38154.800	185 / 500	Yes
B12	primary	2000 / 500	260902	-38261.396	199 / 500	Yes
B13	confirmation	5000 / 1000	260903	-38223.363	41 / 1000	Yes
B14	primary	2000 / 500	260902	-38228.931	196 / 500	Yes

Note. Retention is the percentage of each baseline modal profile assigned to its matched sensitivity profile. All selected runs were admissible and replicated their best likelihood. The selected B13 confirmation used a different seed and increased starts. No convergence or local maximum warnings were recorded for these selected runs. BA = Broadly Adaptive; DD = Depleted-Disconnected; ST = Struggling; UF = Unfulfilled; ES = Engaged-Strained.

Figure S1 Information criteria across S3 profile solutions

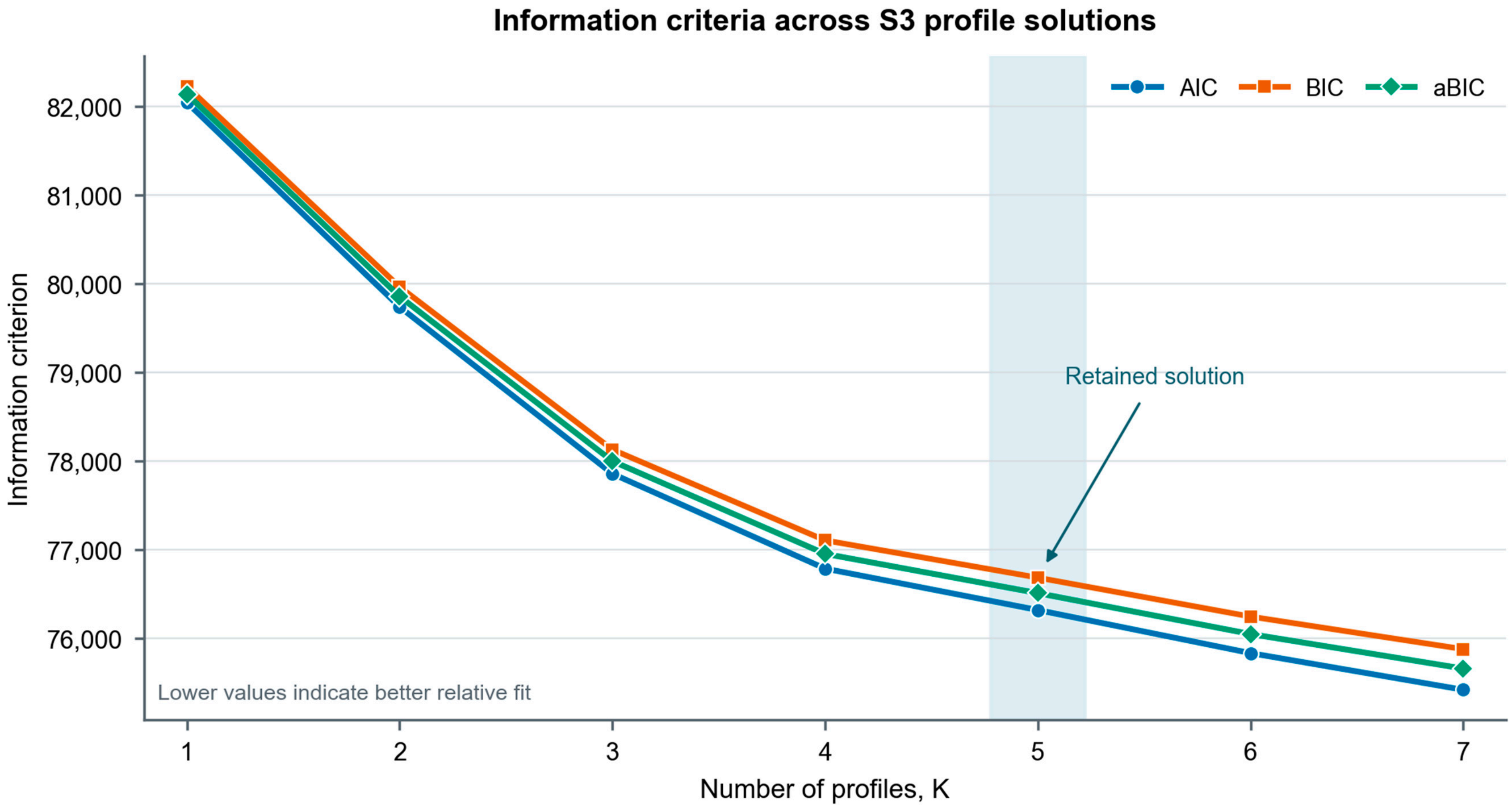

Note. AIC, BIC, and aBIC decreased through K7. The shaded K5 solution was retained after considering class configurations and the additional subdivision at K6. The figure does not identify K5 as the numerical minimum of the information criteria.

Figure S2 Profile configurations following PA item deletion

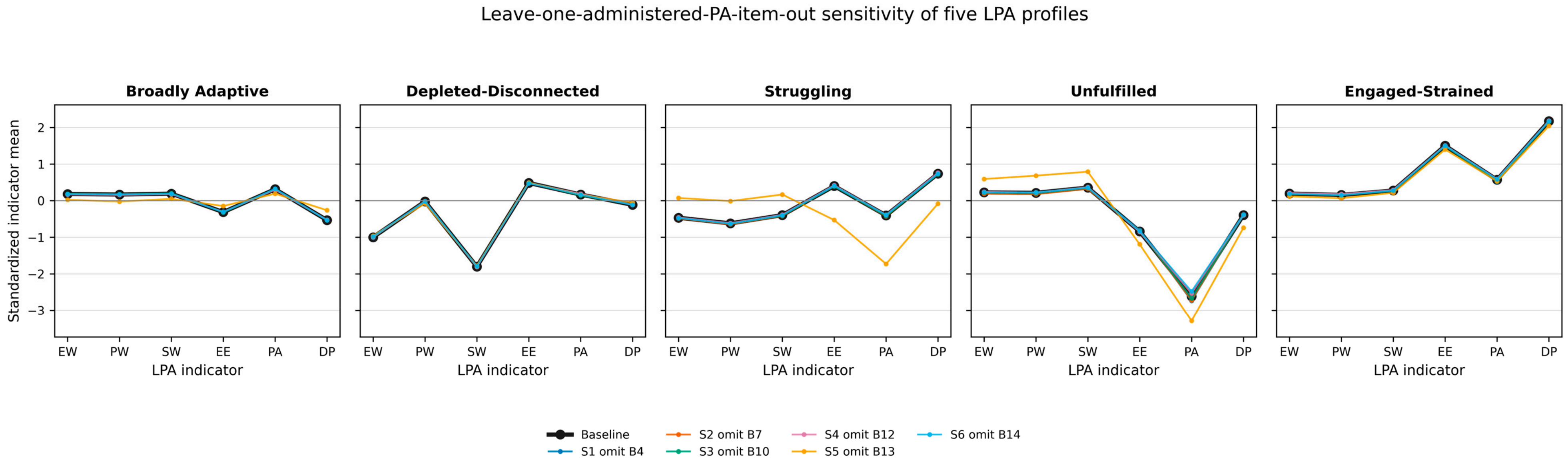

Note. Each panel compares one matched profile in the baseline model and the six PA item deletion models. The vertical axis shows model-estimated standardized indicator means. The gold line denotes deletion of B13. Labels beginning with S1 through S6 in this figure identify the six item deletion runs, not the six variance and covariance specifications in Table S3. Every item deletion model used specification S3 with five profiles. Profile matching after B13 deletion does not establish recovery of the original substantive configuration. EW = emotional well-being; PW = psychological well-being; SW = social well-being; EE = emotional exhaustion; PA = personal accomplishment; DP = depersonalization.
